# Driver Genes Associated With the Incidence of Venous Thromboembolism in Patients With Non-Small-Cell Lung Cancer: A Systematic Review and Meta-Analysis

**DOI:** 10.3389/fonc.2021.680191

**Published:** 2021-04-29

**Authors:** Xiaohan Qian, Mengjiao Fu, Jing Zheng, Jianya Zhou, Jianying Zhou

**Affiliations:** Department of Respiratory Disease, Thoracic Disease Center, The First Affiliated Hospital, Zhejiang University School of Medicine, Hangzhou, China

**Keywords:** venous thromboembolism (VTE), arterial thromboembolism (ATE), non-small-cell lung cancer, *ALK*, *ROS1*, *EGFR*, *KRAS*

## Abstract

**Background:**

The association between driver genes and the incidence of thromboembolic events (TEs) in patients diagnosed with non-small-cell lung cancer (NSCLC) needs to be quantified to guide clinical management.

**Methods:**

We interrogated PubMed, Embase, Web of Science and Cochrane library databases for terms related to venous thromboembolism (VTE) and arterial thromboembolism (ATE) in patients diagnosed with non-small-cell lung cancer harboring driver genes. This search was conducted for studies published between 1 January, 2000 and 31 December, 2020. A random-effects meta-analysis was performed to analyze the pooled incidence and odds ratios of VTE in patients with different driver genes.

**Results:**

Of the 2,742 citations identified, a total of 25 studies that included 21,156 patients met eligibility criteria. The overall pooled incidence of VTE in patients with driver genes was 23% (95% CI 18-29). Patients with *ROS1* rearrangements had the highest incidence of VTE (37%, 95%CI 23-52). *ALK* rearrangements were associated with increased VTE risks (OR=2.08,95% CI 1.69-2.55), with the second highest incidence of VTE (27%, 95%CI 20-35). Both groups of patients with *EGFR* and *KRAS* mutations did not show a significantly increased risk for VTE (OR=1.33, 95% CI 0.75-2.34; OR=1.31, 95% CI 0.40-4.28).

**Conclusions:**

*ALK* rearrangements were shown to be associated with increased VTE risks in patients diagnosed with non-small lung cancer, while there was no significant relation observed between VTE risks and *EGFR* or *KRAS* mutations in lung cancer patients.

## Introduction

Venous thromboembolisms (VTEs), which consist of deep vein thrombosis (DVT) and pulmonary embolisms (PEs), are a common complication associated with cancer, occurring in 5-10% of cancer patients. VTEs are also a major cause of morbidity and mortality (1, 2), increasing risks of death 3-5 times (3). Compared with other malignancies, lung cancer has been associated with an intermediate risk of VTE, especially during the first year following cancer diagnosis. The incidence of VTE in lung cancer patients is approximately 7-13% (1, 4). With prolonged survival, the aging of the cancer population and the introduction of thrombogenic anti-cancer treatments increase the incidence of VTE in cancer patients (2). In addition, patients diagnosed with lung cancer are at an increased risk for arterial thromboembolism (ATE), but the impact on the generation of ATE is less severe than that on the generation of VTE (5).

Molecular subtypes are highly relevant to the outcomes of patients with advanced or metastatic NSCLC, as targeted therapy has become a standard clinical management of patients with specific activating mutations in recent decades, and it can significantly improve the survival of patients. Guidelines recommend that all patients with advanced or metastatic NSCLC should be tested for targetable driver genes as a means to guide treatments (6, 7). Since different driver genes show heterogeneity in tumor biological behavior, treatment response and prognosis, driver genes may also manifest heterogeneity during thrombus generation. Many articles have suggested that *ROS1* and *ALK* rearrangements cause a greater risk for VTE (8–10), but there are still some controversies for patients with *EGFR*, *KRAS* and other gene mutations (8, 11–15). Moreover, there is currently a lack of quantitative analysis determining the extent to which different driver genes affect VTE occurrence, as the incidence has been shown to vary between studies.

Since the primary prevention of VTE in cancer patients is not a validated management strategy, VTE risk assessment tools are necessary for efficient prevention (16). Although there are many clinical scoring systems incorporating known tumor-related VTE risk factors into the scoring system, these tools show poor performance in lung cancer patients (17, 18). Recently, two large random clinical trials investigating direct oral anticoagulants used the Khorana risk scoring system to screen patients in high risks of developing VTE. Results showed that every three VTE cases was prevented at the cost of a major bleeding event caused by thromboprophylaxis (19, 20). Therefore, a VTE risk assessment tool applicable for lung cancer patients is urgently needed. Incorporating positive driver genes into a risk assessment model may improve performance, and thus clarification of the correlation between driver genes and VTE risk is of great significance for VTE risk assessments. Consequently, we generated this systematic review and meta-analysis to evaluate the estimated incidence and risk of VTE in NSCLC patients with different driver genes.

## Methods

### Search Strategies and Selection Criteria

According to the Preferred Reporting Items for Systematic Reviews and Meta-Analyses (PRISMA) statements, we identified relevant studies for meta-analysis and systematic review (**Supplementary Table 1**) (21). We performed a search of PubMed, Embase, Web of Science and Cochrane library databases for relevant articles written in English and published between 1 January, 2000 and 31 December, 2020. We used three groups of search terms including: (1) ‘lung cancer’, ‘lung neoplasms’ and ‘non-small-cell lung cancer’; (2) ‘*EGFR*’, ‘*KRAS*’, ‘*ALK*’, ‘*ROS1*’, ‘*RET*’, ‘*MET*’, ‘*BRAF*’, ‘*MEK*’, ‘*PIK3CA*’, ‘*PTEN*’, ‘*FGFR1*’, ‘*HER2*’ and ‘*DDR2*’; (3) ‘venous thromboembolism’, ‘deep vein thrombosis’, ‘pulmonary embolism’, ‘arterial thromboembolism’, ‘cerebral vascular accident’, ‘stroke’ and ‘myocardial infarction’ (specific search strategies are listed in Supplementary File 1). References of the included studies, published meta-analyses and systematic reviews were also assessed for further potential studies that could be included.

We included all full-text studies and abstracts with information on the incidence of VTE or ATE in lung cancer patients with confirmed driver genes. These studies included hospital-, population- and registry-based cohorts and case-control studies. The inclusion criteria were defined as follows: (1) patients diagnosed with pathologically confirmed NSCLC, (2) a confirmed gene status and (3) available data on VTEs or ATEs. Follow-up duration and treatment regimens were not restricted in any form. The exclusion criteria were defined as follows: (1) clinical trials and other studies on treatment safety (i.e., showing VTE or ATE as adverse events), were excluded due to their miscellaneous follow-up period which is usually not related to the time of cancer diagnosis, (2) case reports, case series, reviews, *in vitro* studies and animal studies. Three investigators independently assessed all trials for eligibility, and disagreements were resolved through consensus.

### Data Extraction

Two independent investigators reviewed titles and abstracts of potentially relevant studies and independently extracted study details using a standardized pilot-tested form. A third investigator reviewed all data entries. The following information from each eligible study was extracted, including: first author, study location, study design, sample size, pathological type, gene status, treatment regimens and follow-up duration. We extracted data on the incidence of VTE and ATE in NSCLC patients. Possible related factors for the development of VTE in NSCLC patients were also extracted when available, including age, sex, ethnicity, pathological type, cancer stage, smoking status, comorbidities, gene subtypes and treatment regimens.

The methodological quality of studies was assessed by two authors using the Newcastle-Ottawa quality assessment scale (22), which assigns 4 points for selection, 2 points for comparability and 3 points for outcomes.

### Outcomes

The primary outcome was the occurrence of VTE, including events during the peri-diagnosis and treatment periods. VTE consists of DVT (symptomatic or asymptomatic) and PE. Secondary outcome was the incidence of ATE. ATE was defined as arterial thromboembolism, including but not limited to cerebral vascular accident and acute myocardial infarction.

### Statistical Analysis

Statistical analyses for overall risks of VTEs/ATEs were performed using Stata 14.0. Study heterogeneity was estimated using the χ^2^-based Q statistic and heterogeneity was considered statistically significant when *I^2^* > 50%. Meta-analysis was performed using the Mantel-Haenszel random-effects model for estimates of the odds ratio (OR) and the inverse variance random-effects model for rate estimates. The association of potential risk factors with VTEs/ATEs was summarized as OR (95% confidence interval [CI]). Given that differences in follow-up periods, the inclusion of asymptomatic VTE, VTE composition, stage, pathological type, race, quality and publish year would affect the result, we performed subgroup analysis based on these factors. Publication bias was assessed through graphical visualization of funnel plots as well as using Begg’s and Egger’s tests. Sensitivity analysis of the primary outcome was conducted by sequential removal of each involved trial. A two-sided *P* value less than 0.05 was considered to be statistically significant.

## Results

**Figure 1:**
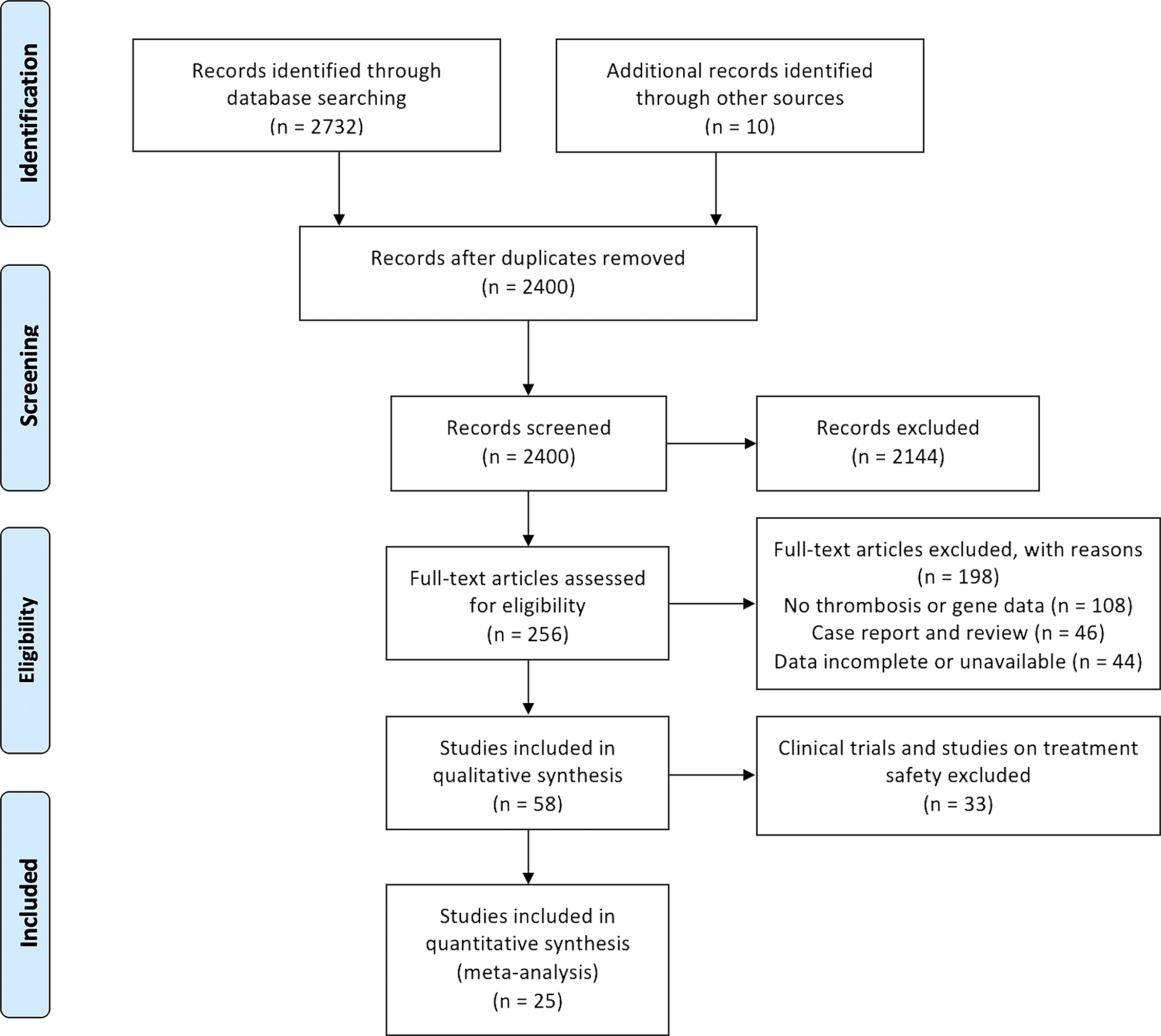
PRISMA flow diagram.

We identified 2,742 reports and included 25 studies performed between January 1, 2000 and December 31, 2020 in the analysis. The process of identifying eligible studies for our systematic review and meta-analysis is shown in **Figure 1**. Characteristics of included studies are available in **Table 1**. 25 studies included 3 prospective cohorts, 20 retrospective cohorts and 2 retrospective case-control studies. Of the 25 studies, 22 studies reported VTEs, and 8 studies reported ATEs. 4 studies reported PEs or DVT events independently. The size of the studies varied between 26 and 4,752 participants. All included studies contained patients diagnosed with NSCLC or lung adenocarcinoma harboring driver genes including *EGFR*, *ALK*, *ROS1*, *KRAS*, *MET* and *BRAF*. All included studies were of good quality, as assessed using the Newcastle-Ottawa quality assessment scale for cohort and case-control studies (**Supplementary Table 2**).

**Table 1:** Characteristics of studies included in the meta-analysis.

Study	Study design *	Country	No. of patients	Stage	Histology	Gene status	TE type	Median follow-up duration/month (range)
Al-Samkari H 2020 (9)	Cohort R	USA	807	III IV	NSCLC	*ALK*	VTE, ATE, PE, DVT	31.5
Alexander M 2020 (23)	Cohort R	Australia	42	NR	NSCLC	*ROS1*	VTE, ATE, PE, DVT	10.9(0.1-180.4)
Azevedo S 2017 § (24)	Cohort R	Portugal	26	NR	NSCLC	*ALK*	VTE, PE	13.5
Berger N 2014 § (25)	Cohort R	USA	57	III IV	AC	*EGFR*	VTE	NR
Chiari R 2020 (10)	Cohort P	Italy	74	IIIB IV	NSCLC	*MET, ROS1*	VTE, ATE, PE, DVT	36.4
Corrales-Rodriguez L 2014 (11)	Case-control R	Canada	159	I-IV	NSCLC	*EGFR, KRAS*	VTE	NR
Davidsson E 2017 (26)	Cohort R	Swidden	310	I-IV	AC	*ALK, EGFR*	VTE	0.3-105.3†
Delmonte A 2015 § (27)	Cohort R	Italy	289	IIIB IV	AC	*ALK, BRAF, EGFR, KRAS*	VTE	NR
Dou F 2018 (13)	Cohort P	China	605	I-IV	NSCLC	*EGFR, KRAS*	VTE	NR
Dou F 2020 (28)	Cohort P	China	341	I-IV	NSCLC	*ALK*	VTE	7.5(3.1-15.4)
Leader A 2019 § (29)	Cohort R	Israeli	4752	NR	NSCLC	*ALK*	ATE	18(12.93-18)
Lee Y G 2014 (30)	Cohort R	Korea	1998	I-IV	NSCLC	*ALK, EGFR*	VTE	45.6
Muñoz-Unceta N 2020 (31)	Cohort R	Spain, Portugal	58	III IV	NSCLC	*ROS1*	VTE, ATE, PE, DVT	19 (1–78)
Ng T L 2019 (8)	Cohort R	China, USA	740	I-IV	NSCLC	*ALK, EGFR, KRAS, ROS1*	VTE, ATE	19.9
Roopkumar J 2020 (32)	Cohort R	USA	461	I-IV	NSCLC	*ALK, EGFR*	PE, DVT	33.1 (0.1-192.4)
Shahzad H 2017 § (33)	Cohort R	USA	62	I-IV	AC	*EGFR*	VTE	NR
Shen Q 2017 (34)	Case-control R	China	1560	III IV	NSCLC	*ALK, EGFR, ROS1*	VTE	NR
Verso M 2015 (12)	Cohort R	Italy	173	IIIB IV	AC	*ALK, EGFR, KRAS*	PE	16.9 ± 8.1 ‡
Wang J 2019 (14)	Cohort R	China	323	NR	AC	*ALK, EGFR, KRAS*	VTE	NR
Xiong W 2020 (35)	Cohort R	China	1187	I-IV	NSCLC	*ALK, BRAF, EGFR, ROS1*	PE	NR
Yamazaki S 2013 § (36)	Cohort R	Japan	1953	NR	NSCLC	*EGFR*	PE	NR
Yang S 2020 (37)	Cohort R	China	513	IIIB IV	AC	*ALK, EGFR*	VTE	30
Zer A 2017 (38)	Cohort R	Canada, Israeli	98	I-IV	AC	*ALK*	VTE, ATE, PE, DVT	22 (1–139)
Zer A 2019 § (39)	Cohort R	Israeli	4327	NR	NSCLC	*ALK*	VTE	NR
Zugazagoitia J 2018 (40)	Cohort R	Spain, Portugal	241	III IV	NSCLC	*ALK*	VTE, ATE, PE	19(0-59)

AC, adenocarcinoma; ATE, arterial thromboembolism; DVT, deep vein thrombosis; NR, not reported; NSCLC, non-small-cell lung cancer; PE, pulmonary embolism; TE, thromboembolic event; VTE, venous thromboembolism.

*R/P stands for Retrospective (R) or prospective (P).

† Follow-up range.

‡ Mean follow-up duration (mean ± standard deviation).

§ Abstract only.

### The Association Between VTE and Driver Genes

**Figure 2:**
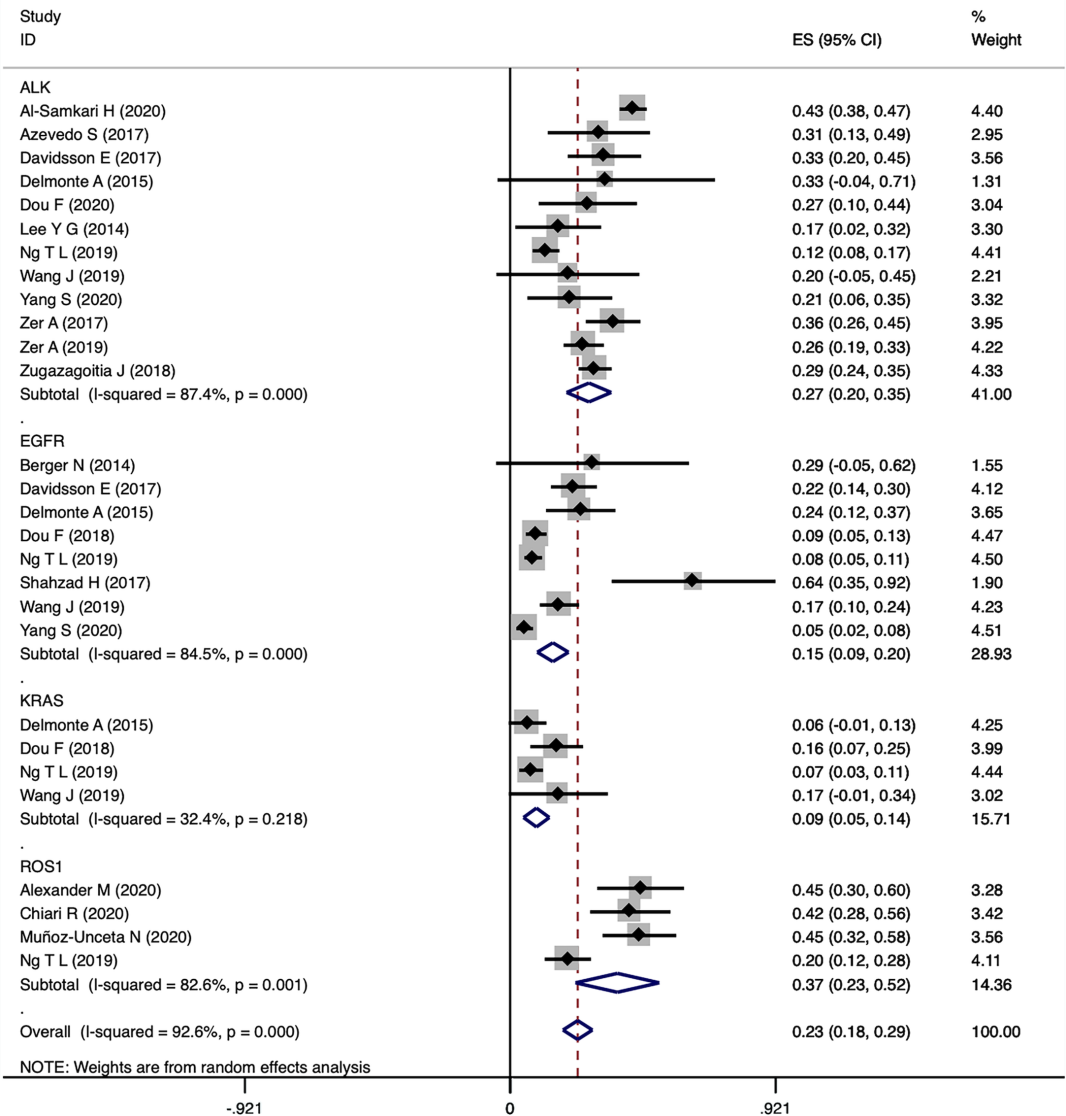
Pooled estimates for incidence of VTE in patients with 4 driver gene types.

**Figure 3:**
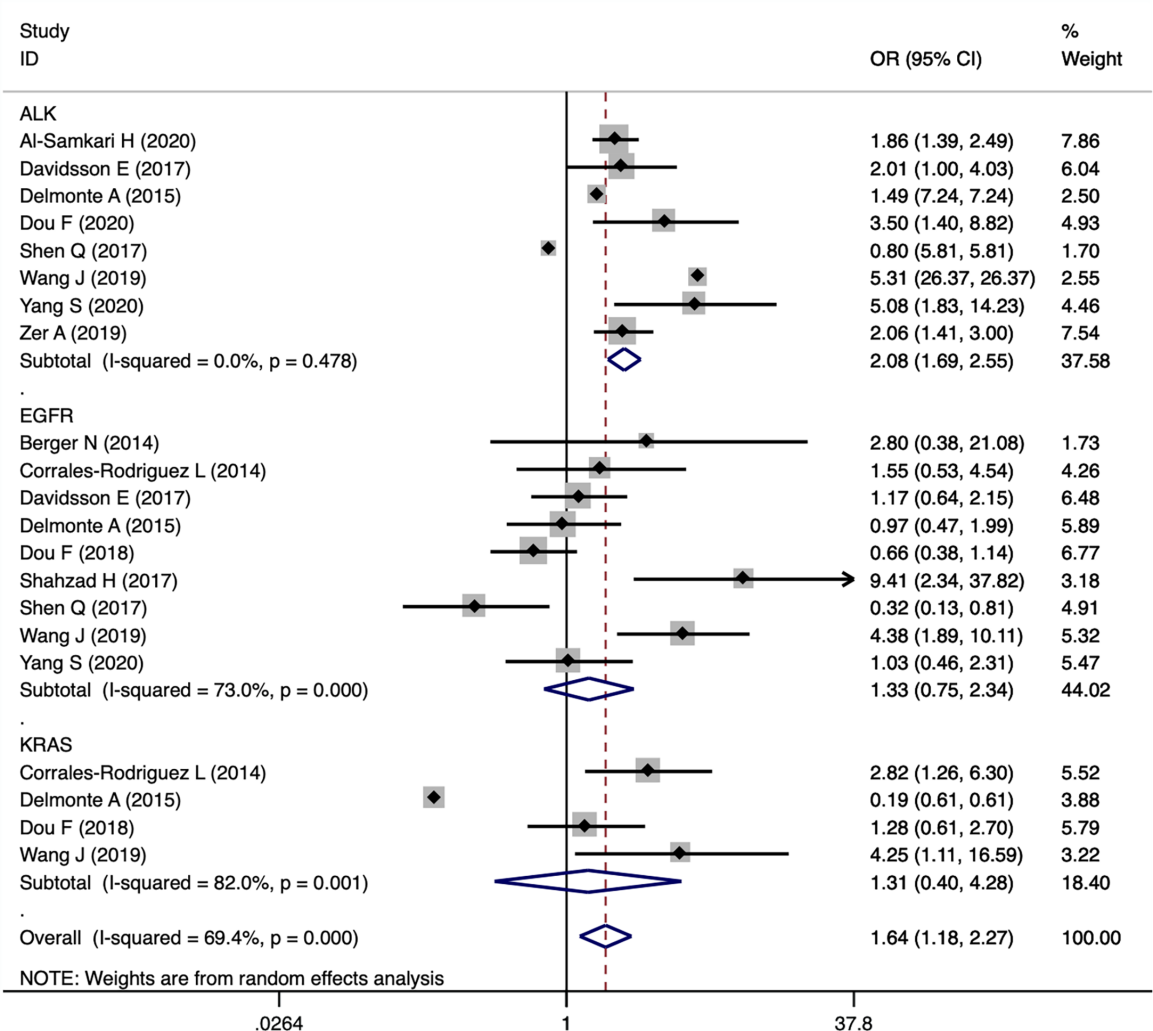
Forest plot demonstrating the association of driver genes with VTE events.

The study included 21,156 patients of which 4,342 patients were confirmed to harbor driver gene mutations (2,080 *EGFR* mutated, 1,575 *ALK* rearranged, 340 *KRAS* mutated, 290 *ROS1* rearranged, 31 *BRAF* mutated and 26 *MET* amplificated or exon 14 mutated; Clinical characteristics of each group with different driver genes were shown in **Supplementary Table 3**). Among the patients, 876 patients developed thromboembolic events, including 630 VTE in 2,882 patients with driver genes and 105 ATE events in 1,821 patients harboring driver genes. Pooled VTE incidence was 23% (95% CI 18-29). Patients with *ROS1* rearrangements had the highest VTE incidence of 37% (95% CI 23-52), followed by *ALK* rearranged patients with an incidence of 27% (95%CI 20-35). VTE incidence in patients with *EGFR* mutations and *KRAS* mutations was 15% (95% CI 9-20) and 9% (95% CI 5-14), respectively (**Figure 2**). Furthermore, 10 studies were analyzed with negative controls as shown in **Figure 3**. Patients with *ALK* rearrangements showed significantly higher risks for VTE (OR=2.08,95% CI 1.69-2.55, *P*=0.000, I^2 =^ 0.0%), while patients harboring *EGFR* (OR=1.33, 95% CI 0.75-2.34, *P*=0.328, I^2 =^ 73.0%) and *KRAS* (OR=1.31, 95% CI 0.40-4.28, *P*=0.652, I^2 =^ 82.0%) mutations showed no significant relation with the risk of VTE.

**Figure 4:**
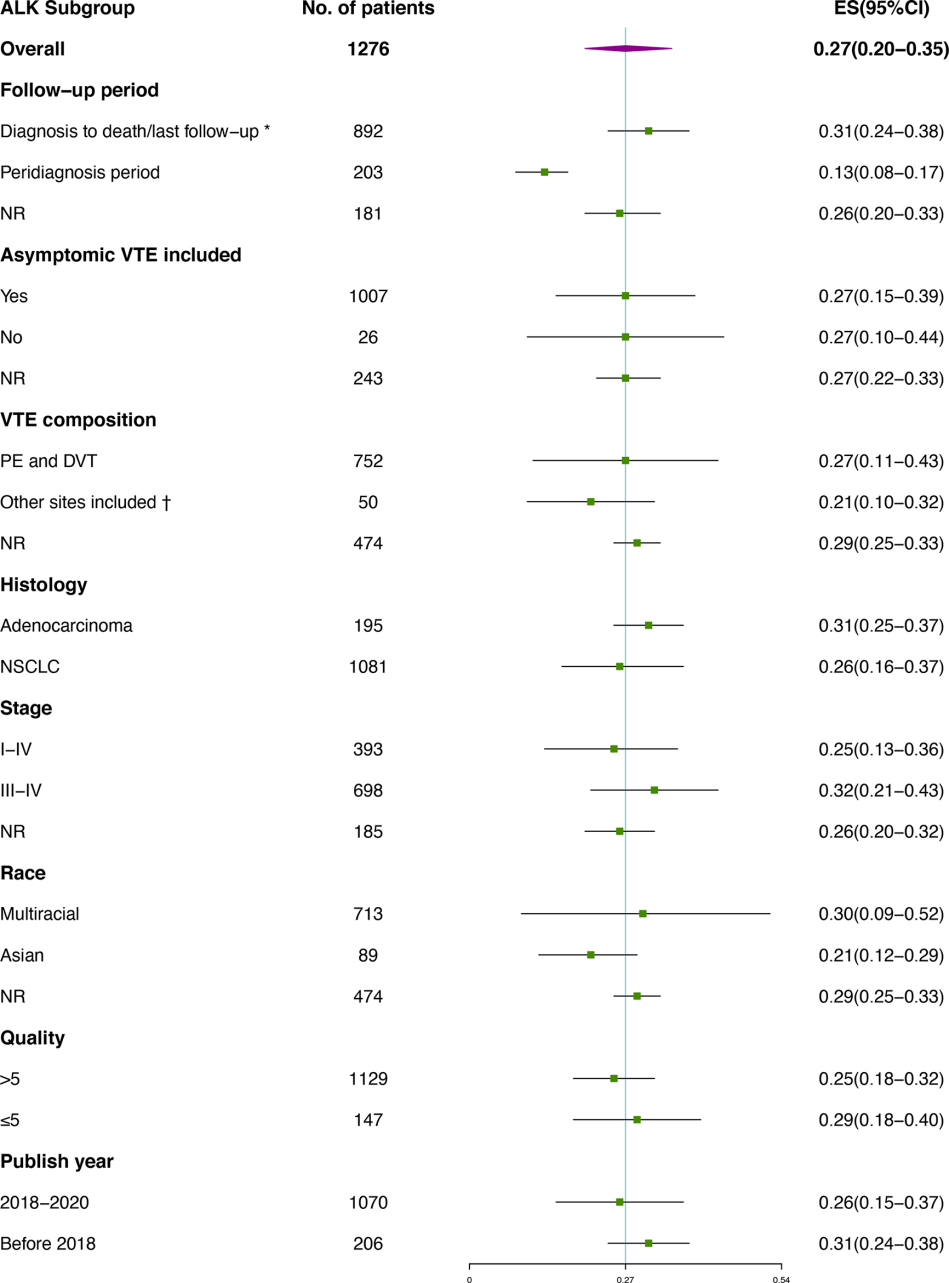
Subgroup analysis of VTE incidence in *ALK* rearranged patients. *Studies containing VTEs that occurred prior to diagnosis were also included; † Besides PE and DVT, venous thrombosis occurred at other sites were also included, including vena cava, neck vein, portal vein and other sites. No catheter-related events was included.

**Figure 5:**
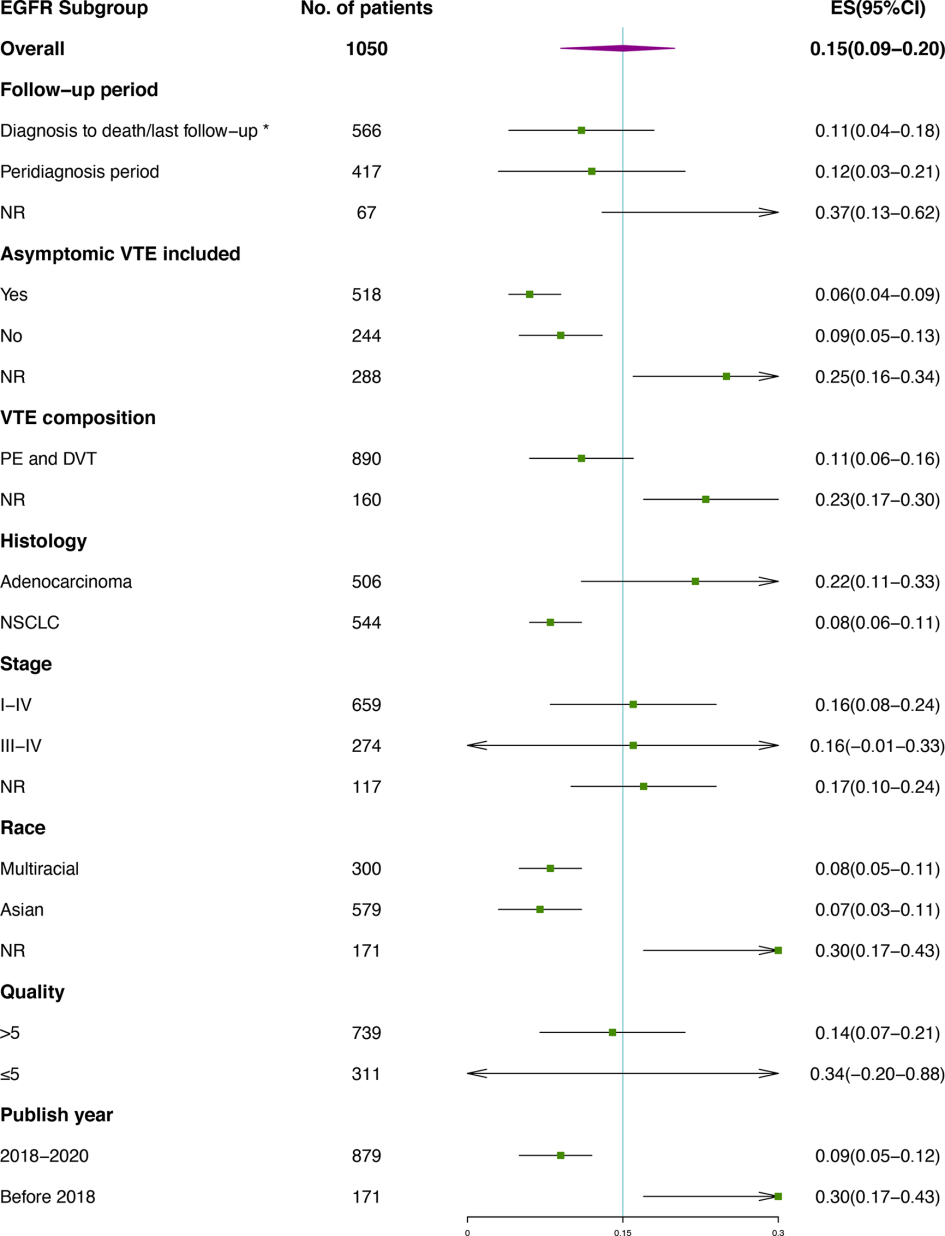
Subgroup analysis of VTE incidence in *EGFR* mutant patients. *Studies containing VTEs that occurred prior to diagnosis were also included.

Subgroup analysis of VTE incidence in *ALK* rearranged or *EGFR* mutant patients (**Figures 4**, **5**) was performed. Great consistency was shown in patients with *ALK* rearrangement. As for the VTE incidence in *EGFR* mutant patients, publish year and insufficient data may explain the heterogeneity of results. There were 5 included studies published before 2018, and 3 of them were conference abstracts with limited information. Other factors including the setting of the follow-up period, the inclusion of asymptomatic VTE, the composition of VTE, histology, stage, race and quality of studies were not significantly associated with the outcomes.

**Figure 6:**
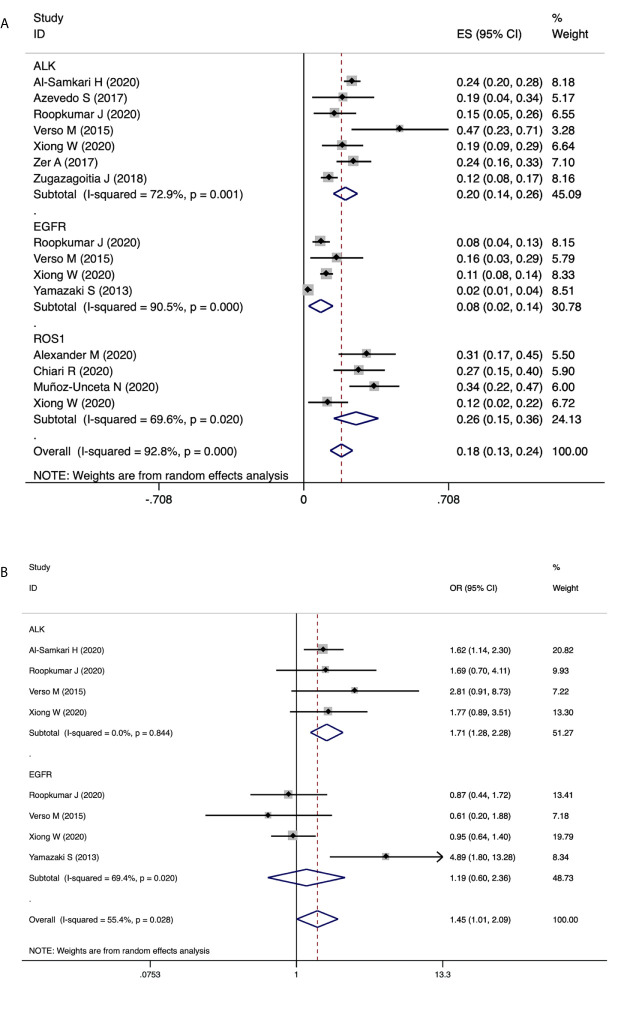
The association of driver genes with PE events. **(A)** Pooled estimates for incidence of PE in patients with *EGFR* mutation, *ALK* rearrangement and *ROS1* rearrangement. **(B)** Forest plot demonstrating the association of driver genes with PE events.

Results of PEs in patients carrying driver genes showed similar trends. As shown in **Figure 6A**, Patients with *ROS1* rearrangements had the highest PE incidence of 26% (95% CI 15-36), followed by *ALK* rearranged patients with an incidence of 20% (95%CI 14-26). PE incidence in patients with *EGFR* mutations was 8% (95% CI 2-14). Furthermore, as shown in **Figure 6B**, patients with *ALK* rearrangements showed significantly higher risks for PE (OR=1.71, 95% CI 1.28-2.28, *P*=0.000, I^2 =^ 0.0%), while patients harboring *EGFR* mutations showed no association with the risk of PE (OR=1.19, 95% CI 0.60-2.36, *P*=0.614, I^2 =^ 69.4%). Sensitivity analysis and publication bias are presented in supplementary figures. No significant publication bias was observed in funnel plots as well as the Egger’s and Begg’s test results.

### The Association Between ATE and Driver Genes

**Figure 7:**
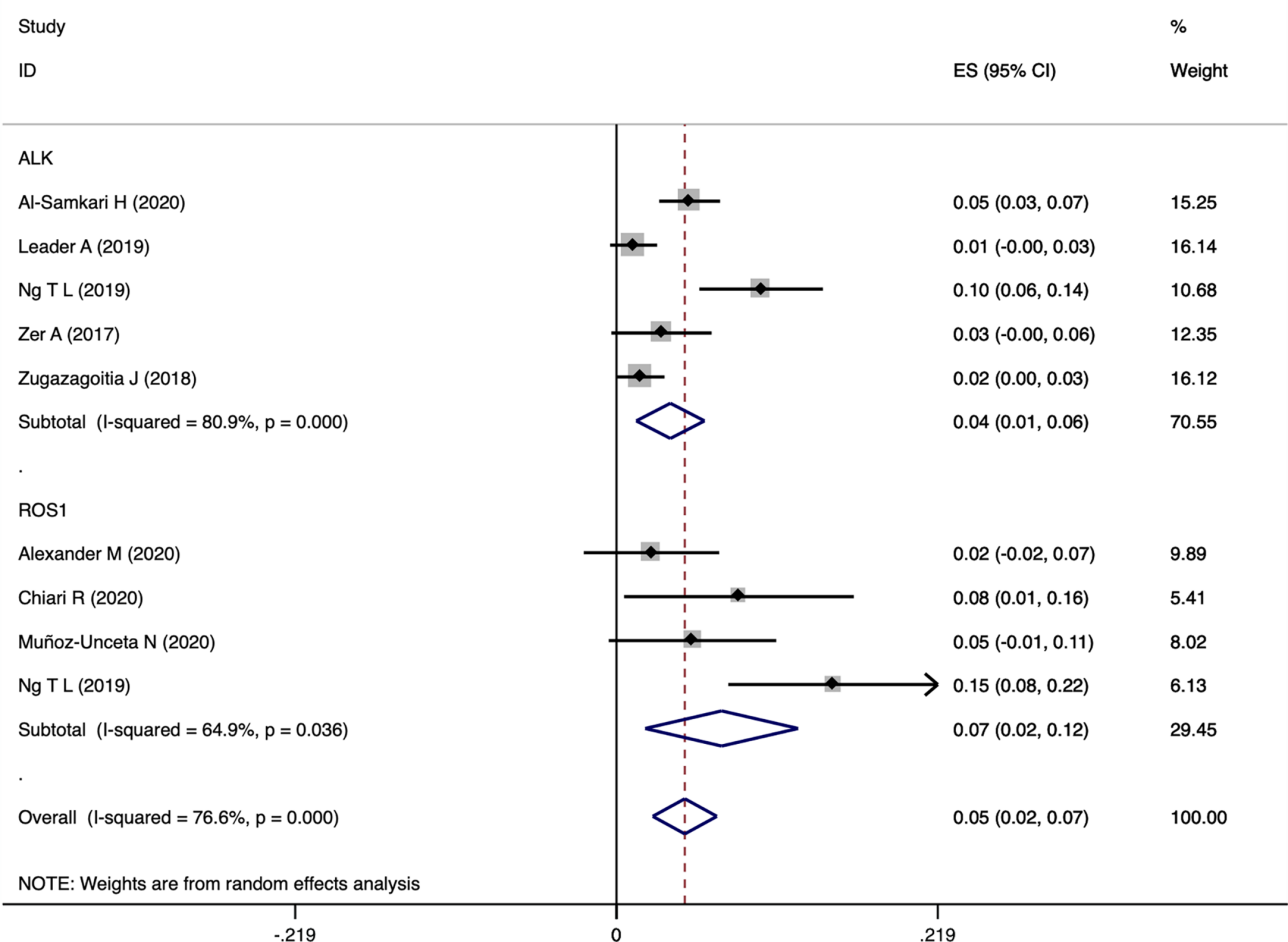
Pooled rate estimates for ATE in patients with *ALK* or *ROS1* rearrangement.

A total of 8 articles recorded ATE incidence in patients with different driver genes, as shown in **Figure 7**. Patients harboring *ROS1* rearrangements showed higher incidence of ATE (7%, 95%CI 2-12), compared with that of *ALK* rearranged patients (4%, 95%CI 1-6). The results were similar to the ATE incidence observed in general lung cancer populations (41). There were only two studies available for risk estimates of ATE in patients with *ALK* rearrangement (9, 29), with a total of 5,289 patients (594 *ALK* rearranged). The results showed that *ALK* rearrangement did not significantly increase the risk of ATE (OR=0.92, 95% CI 0.44-1.92, *P*=0.823, I^2 =^ 19.4%).

## Discussion

Even though recent articles indicate that both *ROS1* and *ALK* rearrangements are associated with a higher risk for VTE, the incidence and relative risks reported vary between studies. In addition, there is controversy regarding the correlation between *EGFR* or *KRAS* mutations and VTE risks. In this systematic review and meta-analysis, we analyzed VTE incidence as well as the estimated risk for VTE in lung cancer patients with driver genes including *ROS1*, *ALK*, *EGFR* and *KRAS*. Patients with *ROS1* and *ALK* rearrangements showed the highest incidence for VTE, and the risk of VTE in *ALK* rearranged patients was double of that observed in *ALK* wild type patients. Patients with *KRAS* mutations showed the lowest incidence for VTE among the analyzed driver genes, and both *KRAS* and *EGFR* mutations were proved no significant relation with VTE risks in patients with lung cancer. These findings suggest that incorporating *ROS1* and *ALK* rearrangements into VTE risk assessment models may be beneficial for screening NSCLC patients with a high risk for VTE.

Among patients with driver gene mutations, the incidence of pooled VTE was 23% (95% CI 18-29), which was higher than the reported incidence of 7-13% in lung cancer patients (1, 4). This discrepancy may attribute to the difference of population compositions. In the general population, patients with *ALK* and *ROS1* rearrangements are observed in 5% and 2% of NSCLC patients, respectively (42, 43). In the population with driver gene mutations studied in this article, patients with *ALK* rearrangements accounted for 29.6% while patients with *ROS1* rearrangements accounted for 8.4%. Both of these proportions were much greater than what was observed in the general population. Given that patients with *ROS1* and *ALK* rearrangements show longer survival rates compared to patients with other driver genes, several studies performed survival analyses to prove that the increased risk of VTE was not due to prolonged survival (8, 9, 28, 40). Consistent with previous studies, VTE that occurred in the peri-diagnostic period (one month prior to and after cancer diagnosis) accounted for 32%~45% (10, 23) and 23%~35% (9, 38, 40) of all VTEs in the *ROS1* or *ALK* positive cohort, respectively.

There is no doubt that the major purpose of genetic testing is to guide treatment, and NSCLC with different driver genes has different optimal treatment options according to the guidelines (7). As the studies included in this meta-analysis provided limited treatment information (as shown in **Supplementary Table 3**), and preferred treatments for driven genes has been changing rapidly within years, it becomes quite difficult to diminish the treatment related bias through analysis. However, detailed information from several studies included confirmed the relatively higher risks of TE in NSCLC with *ALK/ROS1* rearrangements regardless of the effects caused by treatments. When the timing of TE events was analyzed, 50%~59% of TE in *ROS1* rearranged patients happened with naïve treatments (23, 31), while 37% of VTE in patients with *ALK* rearrangements occurred when patients received no treatments (38). In a study comparing the risk of TE between *EGFR* mutant and *ALK* rearranged patients, the incidence of TE before using TKI and the incidence of TE in patients who have never used TKI in patients with *ALK* rearrangements were significantly higher than those in patients with *EGFR* mutations (50% vs 17.2%; 90.9% vs 21.2%, respectively). The study also found that using TKI before thrombosis is a protective factor for TEs in *ALK* rearranged patients [HR=0.084 (95%CI 0.031-0.232)], which was not observed in *EGFR* mutant patients. This result suggests that the thrombotic predisposition of *ALK* rearrangement may be related to the gene alteration and derived kinase activity (32). In addition, a recent meta-analysis indicated that TKIs treating NSCLC with *ALK/ROS1* rearrangements did not significantly increase the VTE risk compared with platinum-based chemotherapy by analyzing 6 randomized control studies (44). All the evidence supports that *ALK/ROS1* rearrangements are associated with increased TE risks. None of the included studies mentioned immunotherapy in treatment modalities. With the advancement of treatments and the development of precise management for target genes in the future, the incidence of TE during clinical management in patients with different driver genes may change accordingly. However, putting aside the influence of treatments on TE events, it is unquestionable that driver genes play an important role in the occurrence of TE events.

The incidence of ATE in patients with lung cancer is approximately 6.3%-10.9% (5, 41). Studies have suggested that the risk of ATE may be the greatest during the peri-diagnosis period, which is similar to results in VTE studies (5, 45, 46). A recent study retrospectively analyzed an *ALK* positive cohort in NSCLC patients. Although the incidence of ATE was similar in patients with and without *ALK* rearrangements (5.0% vs 4.4%), *ALK*-positive patients showed a 3-fold greater risk than wild type patients using time-to-event analysis with relevant variables adjusted (9). Therefore, even though the incidence and risk estimate of ATE in patients with *ALK* or *ROS1* rearrangement in our study were not significantly higher than that in the general lung cancer population, harboring driver genes such as *ALK*, may increase the risk of ATE generation in a short period after diagnosis with lung cancer. However, current research data for the relation between ATE and driver genes in cancer patients were not strong enough to support the hypothesis. More prospective studies with time-to-event information are expected.

Underlying molecular mechanisms of tumor genomic mutations affecting thrombosis are still unclear. Tissue factor (TF) is an important physiological trigger of coagulation. Upregulation in TF may contribute to the prethrombotic state linked to malignant tumors (47, 48). *ALK* rearrangements have been shown to be associated with high levels of TF (49). *KRAS* mutations have also been associated with increased TF expression levels in colorectal cancer and NSCLC (50–52). Moreover, it is known that inflammation plays an important role in thrombosis induction (53). Some studies on *ALK*-mutated lymphomas have shown that *ALK* rearrangements lead to increased STAT3 signal transduction, which is involved in downstream signaling of inflammatory cytokines (54). It has also been shown that ALK is important in the activation of NLRP3 inflammasomes in macrophages (55). From a macroscopic perspective, the thrombosis risk assessment model, ONKOTEV study included vascular or lymphatic macroscopic compression as one of the assessment criteria, suggesting that vascular or lymphatic compression may be related to increased VTE risk (56). Studies have shown that higher N stage is associated with an increased VTE risk (57). *ALK* and *ROS1* are common fusion genes in NSCLC, with similar histological findings (58) and a higher tendency to lymph node metastasis (59, 60). In this study, patients with *ALK* rearrangements showed a higher risk of VTE, while patients with *ROS1* rearrangements showed the highest incidence of VTE. Although the mechanism is not clear yet, the tendency of fusion genes to lymph node metastases may be related to increased risk of VTE.

In addition to driver genes, one study included in our study investigated the correlation between programmed cell death ligand 1 (PD-L1) expression and the risk of PE in NSCLC patients. PD-L1 expression is one of the important biomarkers to predict the potential benefit from anti-PD-1/PD-L1 treatment. The study showed that patients with PD-L1 expression in ≥1% tumor cells had a higher risk for PE [OR=1.798 (95%CI 1.137-2.201)], with an incidence of 15.4%, which indicated that PD-L1 expression may be a novel biomarker in prediction of TE in patients with NSCLC (35). Nichetti F et al. also found that high PD-L1 expression (≥50%) was one of the independent predisposing factors of TE during immune checkpoint inhibitor (ICI) treatments in patients with locally advanced or metastatic NSCLC [HR=2.55 (95% CI 1.05-6.19)] (61). However, another study in patients with glioma showed that PD-L1 expression was not related with the risk of VTE (62). At present, both clinical and fundamental researches on the association between PD-1/PD-L1 and TE are limited. With regards to the generation of ATE, it has been shown that the blockade of PD-1 pathway promotes the activation of proatherogenic T cell, thereby aggravating hyperlipidemia and accelerating the formation of atherosclerosis, which suggests that ICI may potentially foster ATEs (63, 64). As for VTEs, it has been reported that activated T cell subsets *in vitro* express functional TF on their cellular membranes (65). In addition, activated lymphocytes can release a variety of pro-inflammatory cytokines that promote the formation of hypercoagulable states as well (66). It is known that PD-L1 plays an important role in regulating T cell-mediated immune response (67). Therefore, when PD-1/PD-L1 pathway is blocked, the activated immune response may promote the occurrence of TE. However, current studies have indicated that the incidence of TE in patients receiving ICI did not increase significantly, and TE events are not considered as an ICIs drug related toxicity commonly (61, 68). Based on current results, we hypothesize that the risk of TE caused by the activated immune response may be more prominent in patients with high PD-L1 expression. PD-L1, as a potential biomarker for the risk of TE, may cover more pathological types other than lung adenocarcinoma, since driver genes are more common in patients with adenocarcinoma.

Despite these findings, there were also some limitations faced in this study. First, included studies varied in histological type, tumor staging, diagnostic methodology, follow-up duration and treatment. The existence of confounding factors may lead to significant heterogeneity for some gene mutations (I^2^>50%). Subgroup analysis was performed to analyze potential confounding factors. Second, for some gene mutations, such as *ROS1*, *KRAS*, *MET* and *BRAF*, there were few published studies, so the results for these mutations may have weaker power. Third, when calculating the estimated risk, the population of wild type was shown to be heterogeneous. For example, patients with *EGFR* wild type may harbor *ROS1* and *ALK* rearrangements as well as other unknown gene mutations. Therefore, the calculated risk may show some deviation.

In conclusion, driver genes were associated with the risk of VTE in patients with NSCLC. Patients with *ROS1* rearrangements showed the highest incidence of VTE with 37% (95%CI 23-52). *ALK* rearrangements caused approximately twice the risk for VTE, compared with patients with *ALK* wild type, and patients with *ALK* arrangements had the second highest incidence of VTE with 27% (95%CI 20-35). Patients with *KRAS* and *EGFR* mutations did not show a significant association with VTE risk in patients with NSCLC.

## Data Availability Statement

The original contributions presented in the study are included in the article/**Supplementary Material**. Further inquiries can be directed to the corresponding author.

## Author Contributions

JyiZ, MF, and XQ conceived of the study. MF obtained funding. The concept and design of this study were generated primarily by XQ. XQ, MF, and JyaZ collected data in the original studies. XQ and JyiZ performed the statistical analyses. XQ and MF interpreted the data and drafted the manuscript. MF and JyaZ revised the manuscript for important intellectual content. All authors contributed to the article and approved the submitted version.

## Funding

This work was supported by grants received from the Health Commission of Zhejiang Province, China (grant number: 2020392137). The funding agencies played no role in the study design, participant recruitment, data collection, data analysis, data interpretation, manuscript preparation and the decision to submit the paper for publication.

## Conflict of Interest

The authors declare that the research was conducted in the absence of any commercial or financial relationships that could be construed as a potential conflict of interest.

## Abbreviation

AC, adenocarcinoma; ATE, arterial thromboembolism; DVT, deep vein thrombosis; HR, hazard ratio; ICI, immune checkpoint inhibitor; NSCLC, non-small-cell lung cancer; OR, odds ratio; PD-1, programmed cell death-1; PD-L1, programmed cell death ligand 1; PE, pulmonary embolism; TE, thromboembolic events; TF, tissue factor; VTE, venous thromboembolism.
