# Supplementary material for: Driver Genes Associated With the Incidence of Venous Thromboembolism in Patients With Non-Small-Cell Lung Cancer: A Systematic Review and Meta-Analysis
Source: Front Oncol. 2021 Apr 29;11:680191. doi: 10.3389/fonc.2021.680191 (PMC8117344; doi:10.3389/fonc.2021.680191)
Supplement: Supplementary file 1 [file DataSheet_1.docx]

Supplementary Material

# Supplementary Data

## Supplementary File 1. Search strategy.

### Pubmed search strategy

"Lung Neoplasms"[Mesh] OR "Carcinoma, Non-Small-Cell Lung"[Mesh] OR "Small Cell Lung Carcinoma"[Mesh] OR (Lung cancer*) OR (lung malignan*) OR (lung neoplasm*) OR (lung adenocarcinoma*) OR (lung tumour*) OR (lung tumor*) OR (Neoplasms, Lung) OR (Pulmonary Neoplasm*) OR (Lung Neoplasm*) OR (pulmonary Cancer*) OR (Small Cell Lung Cancer) OR (Oat Cell Lung Cancer) OR (non small cell lung cancer) OR (Non-Small-Cell Lung Carcinomas) OR (Nonsmall Cell Lung Cancer) OR (Non-Small-Cell Lung Carcinoma) OR (Non Small Cell Lung Carcinoma) OR (Non-Small Cell Lung Cancer)

"Genes, erbB-1"[MeSH Terms] OR "Anaplastic Lymphoma Kinase"[MeSH Terms] OR "Proto-Oncogene Proteins p21(ras)"[MeSH Terms] OR "Proto-Oncogene Proteins c-ret"[MeSH Terms] OR "Oncogene Protein tpr-met"[MeSH Terms] OR "Mitogen-Activated Protein Kinase Kinases"[MeSH Terms] OR "PTEN Phosphohydrolase"[MeSH Terms] OR "Receptor, Fibroblast Growth Factor, Type 1"[MeSH Terms] OR "Genes, erbB-2"[MeSH Terms] OR "Proto-Oncogene Proteins B-raf"[MeSH Terms] OR "Discoidin Domain Receptor 2"[MeSH Terms] OR “ROS1 protein, human”[Supplementary Concept] OR EGFR OR ALK OR KRAS OR PIK3CA OR RET OR ROS1 OR MET OR MEK OR PTEN OR FGFR1 OR HER2 OR BRAF OR DDR2

thromboembolism[MeSH Terms]  OR "venous thromboembolism"[MeSH Terms] OR "pulmonary embolism"[MeSH Terms] OR "deep vein thrombosis"[MeSH Terms] OR "Intracranial Embolism and Thrombosis"[Mesh] OR “Stroke"[Mesh] OR “Brain infarction”[Mesh] OR "Myocardial Infarction"[Mesh] OR thromboembolism OR (deep vein thrombosis) OR (pulmonary embolism) OR (venous thromboembolism) OR (Arterial Thromboembolism) OR (Cerebral Vascular Accident*) OR (Cerebral Embolism and Thrombosis) OR (Brain Embolism and Thrombosis) OR (Stroke*) OR (Cerebrovascular Accident*) OR (Cerebrovascular Apoplexy) OR (Brain Vascular Accident*) OR (Cerebrovascular Stroke*) OR Apoplexy OR (Cerebral Stroke*) OR (Acute Stroke*) OR (Acute Cerebrovascular Accident*) OR (Acute Myocardial Infarction) OR (Myocardial Infarct*) OR (Cardiovascular Stroke*) OR (Heart Attack*)

#1 AND #2 AND #3

#4 Filters: Humans

#5 Filters: Publication date from 2000/01/01 to 2020/12/31

#6 Filters: English

### Embase search strategy

Lung cancer*) OR (lung malignan*) OR (lung neoplasm*) OR (lung adenocarcinoma*) OR (lung tumour*) OR (lung tumor*) OR (Neoplasms, Lung) OR (Pulmonary Neoplasm*) OR (Lung Neoplasm*) OR (pulmonary Cancer*) OR (Small Cell Lung Cancer) OR (Oat Cell Lung Cancer) OR (non small cell lung cancer) OR (Non-Small-Cell Lung Carcinomas) OR (Nonsmall Cell Lung Cancer) OR (Non-Small-Cell Lung Carcinoma) OR (Non Small Cell Lung Carcinoma) OR (Non-Small Cell Lung Cancer)

EGFR OR ALK OR KRAS OR PIK3CA OR RET OR ROS1 OR MET OR MEK OR PTEN OR FGFR1 OR HER2 OR BRAF OR DDR2

thromboembolism OR (deep vein thrombosis) OR (pulmonary embolism) OR (venous thromboembolism) OR (Arterial Thromboembolism) OR (Cerebral Vascular Accident*) OR (Cerebral Embolism and Thrombosis) OR (Brain Embolism and Thrombosis) OR (Stroke*) OR (Cerebrovascular Accident*) OR (Cerebrovascular Apoplexy) OR (Brain Vascular Accident*) OR (Cerebrovascular Stroke*) OR Apoplexy OR (Cerebral Stroke*) OR (Acute Stroke*) OR (Acute Cerebrovascular Accident*) OR (Acute Myocardial Infarction) OR (Myocardial Infarct*) OR (Cardiovascular Stroke*) OR (Heart Attack*)

#1 AND #2 AND #3 AND [humans]/lim AND [english]/lim AND[2000-2020]/p

### Web of Science search strategy

TS=((Lung cancer*) OR (lung malignan*) OR (lung neoplasm*) OR (lung adenocarcinoma*) OR (lung tumour*) OR (lung tumor*) OR (Neoplasms, Lung) OR (Pulmonary Neoplasm*) OR (Lung Neoplasm*) OR (pulmonary Cancer*) OR (Small Cell Lung Cancer) OR (Oat Cell Lung Cancer) OR (non small cell lung cancer) OR (Non-Small-Cell Lung Carcinomas) OR (Nonsmall Cell Lung Cancer) OR (Non-Small-Cell Lung Carcinoma) OR (Non Small Cell Lung Carcinoma) OR (Non-Small Cell Lung Cancer))

TS=(EGFR or ALK or KRAS or PIK3CA or RET or ROS1 or MET or MEK or PTEN or FGFR1 or HER2 or BRAF or DDR2)

TS=((Vascular Accident*) OR (Cerebral Embolism and Thrombosis) OR (Brain Embolism and Thrombosis) OR (Stroke*) OR (Cerebrovascular Accident*) OR (Cerebrovascular Apoplexy) OR (Brain Vascular Accident*) OR (Cerebrovascular Stroke*) OR Apoplexy OR (Cerebral Stroke*) OR (Acute Stroke*) OR (Acute Cerebrovascular Accident*) OR (Acute Myocardial Infarction) OR (Myocardial Infarct*) OR (Cardiovascular Stroke*) OR (Heart Attack*))

#3 AND #2 AND #1

Databases= WOS, BCI, CSCD, DIIDW, FSTA, KJD, MEDLINE, RSCI, SCIELO, ZOOREC Timespan=2000-2020

Search language=English

### Cochrane Library search strategy

#1 MeSH descriptor: [Lung Neoplasms] explode all trees
#2 MeSH descriptor: [Carcinoma, Non-Small-Cell Lung] explode all trees
#3 MeSH descriptor: [Small Cell Lung Carcinoma] explode all trees
#4 (Lung cancer*) OR (lung malignan*) OR (lung neoplasm*) OR (lung adenocarcinoma*) OR (lung tumour*) OR (lung tumor*) OR (Neoplasms, Lung) OR (Pulmonary Neoplasm*) OR (Lung Neoplasm*) OR (pulmonary Cancer*) OR (Small Cell Lung Cancer) OR (Oat Cell Lung Cancer) OR (non small cell lung cancer) OR (Non-Small-Cell Lung Carcinomas) OR (Nonsmall Cell Lung Cancer) OR (Non-Small-Cell Lung Carcinoma) OR (Non Small Cell Lung Carcinoma) OR (Non-Small Cell Lung Cancer)
#5 #1 OR #2 OR #3 OR #4
#6 MeSH descriptor: [Genes, erbB-1] explode all trees
#7 MeSH descriptor: [Anaplastic Lymphoma Kinase] explode all trees
#8 MeSH descriptor: [Proto-Oncogene Proteins p21(ras)] explode all trees
#9 MeSH descriptor: [Proto-Oncogene Proteins c-ret] explode all trees
#10 MeSH descriptor: [Oncogene Protein tpr-met] explode all trees
#11 MeSH descriptor: [Mitogen-Activated Protein Kinase Kinases] explode all trees
#12 MeSH descriptor: [PTEN Phosphohydrolase] explode all trees
#13 MeSH descriptor: [Receptor, Fibroblast Growth Factor, Type 1] explode all trees
#14 MeSH descriptor: [Genes, erbB-2] explode all trees
#15 MeSH descriptor: [Proto-Oncogene Proteins B-raf] explode all trees
#16 MeSH descriptor: [Discoidin Domain Receptor 2] explode all trees
#17 EGFR or ALK or KRAS or PIK3CA or RET or ROS1 or MET or MEK or PTEN or FGFR1 or HER2 or BRAF or DDR2
#18 #6 OR #7 OR #8 OR #9 OR #10 OR #11 OR #12 OR #13 OR #14 OR #15 OR #16 OR #17
#19 MeSH descriptor: [Thromboembolism] explode all trees
#20 MeSH descriptor: [Thrombosis] explode all trees
#21 MeSH descriptor: [Pulmonary Embolism] explode all trees
#22 MeSH descriptor: [Stroke] explode all trees
#23 MeSH descriptor: [Myocardial Ischemia] explode all trees
#24 MeSH descriptor: [Brain Infarction] explode all trees
#25 thromboembolism OR (deep vein thrombosis) OR (pulmonary embolism) OR (venous thromboembolism) OR (Arterial Thromboembolism) OR (Cerebral Vascular Accident*) OR (Cerebral Embolism and Thrombosis) OR (Brain Embolism and Thrombosis) OR (Stroke*) OR (Cerebrovascular Accident*) OR (Cerebrovascular Apoplexy) OR (Brain Vascular Accident*) OR (Cerebrovascular Stroke*) OR Apoplexy OR (Cerebral Stroke*) OR (Acute Stroke*) OR (Acute Cerebrovascular Accident*) OR (Acute Myocardial Infarction) OR (Myocardial Infarct*) OR (Cardiovascular Stroke*) OR (Heart Attack*)
#26 #19 OR #20 OR #21 OR #22 OR #23 OR #24 OR #25
#27 #5 AND #18 AND #26 with Cochrane Library publication date Between Jan 2000 and Dec 2020

# Supplementary Tables

**Supplementary Table 1.** PRISMA checklist.

| **Section/topic** | **#** | **Checklist item** | **Reported on page #** |
| --- | --- | --- | --- |
| **TITLE** | | |  |
| Title | 1 | Identify the report as a systematic review, meta-analysis, or both. | #1 |
| **ABSTRACT** | | |  |
| Structured summary | 2 | Provide a structured summary including, as applicable: background; objectives; data sources; study eligibility criteria, participants, and interventions; study appraisal and synthesis methods; results; limitations; conclusions and implications of key findings; systematic review registration number. | #1 |
| **INTRODUCTION** | | |  |
| Rationale | 3 | Describe the rationale for the review in the context of what is already known. | #2 |
| Objectives | 4 | Provide an explicit statement of questions being addressed with reference to participants, interventions, comparisons, outcomes, and study design (PICOS). | #2 |
| **METHODS** | | |  |
| Protocol and registration | 5 | Indicate if a review protocol exists, if and where it can be accessed (e.g., Web address), and, if available, provide registration information including registration number. | NA |
| Eligibility criteria | 6 | Specify study characteristics (e.g., PICOS, length of follow-up) and report characteristics (e.g., years considered, language, publication status) used as criteria for eligibility, giving rationale. | #3 |
| Information sources | 7 | Describe all information sources (e.g., databases with dates of coverage, contact with study authors to identify additional studies) in the search and date last searched. | #2 |
| Search | 8 | Present full electronic search strategy for at least one database, including any limits used, such that it could be repeated. | #2, Appendix |
| Study selection | 9 | State the process for selecting studies (i.e., screening, eligibility, included in systematic review, and, if applicable, included in the meta-analysis). | #3 |
| Data collection process | 10 | Describe method of data extraction from reports (e.g., piloted forms, independently, in duplicate) and any processes for obtaining and confirming data from investigators. | #3 |
| Data items | 11 | List and define all variables for which data were sought (e.g., PICOS, funding sources) and any assumptions and simplifications made. | #3 |
| Risk of bias in individual studies | 12 | Describe methods used for assessing risk of bias of individual studies (including specification of whether this was done at the study or outcome level), and how this information is to be used in any data synthesis. | #3 |
| Summary measures | 13 | State the principal summary measures (e.g., risk ratio, difference in means). | #3 |
| Synthesis of results | 14 | Describe the methods of handling data and combining results of studies, if done, including measures of consistency (e.g., I^2^) for each meta-analysis. | #3 |

Page 1 of 2

| **Section/topic** | **#** | **Checklist item** | **Reported on page #** |
| --- | --- | --- | --- |
| Risk of bias across studies | 15 | Specify any assessment of risk of bias that may affect the cumulative evidence (e.g., publication bias, selective reporting within studies). | #3 |
| Additional analyses | 16 | Describe methods of additional analyses (e.g., sensitivity or subgroup analyses, meta-regression), if done, indicating which were pre-specified. | #3 |
| **RESULTS** | | |  |
| Study selection | 17 | Give numbers of studies screened, assessed for eligibility, and included in the review, with reasons for exclusions at each stage, ideally with a flow diagram. | #4 |
| Study characteristics | 18 | For each study, present characteristics for which data were extracted (e.g., study size, PICOS, follow-up period) and provide the citations. | #4, Table 1 |
| Risk of bias within studies | 19 | Present data on risk of bias of each study and, if available, any outcome level assessment (see item 12). | #4-5 |
| Results of individual studies | 20 | For all outcomes considered (benefits or harms), present, for each study: (a) simple summary data for each intervention group (b) effect estimates and confidence intervals, ideally with a forest plot. | #4-5 |
| Synthesis of results | 21 | Present results of each meta-analysis done, including confidence intervals and measures of consistency. | #4-5 |
| Risk of bias across studies | 22 | Present results of any assessment of risk of bias across studies (see Item 15). | Appendix |
| Additional analysis | 23 | Give results of additional analyses, if done (e.g., sensitivity or subgroup analyses, meta-regression [see Item 16]). | Figure 4-5, Appendix |
| **DISCUSSION** | | |  |
| Summary of evidence | 24 | Summarize the main findings including the strength of evidence for each main outcome; consider their relevance to key groups (e.g., healthcare providers, users, and policy makers). | #5 |
| Limitations | 25 | Discuss limitations at study and outcome level (e.g., risk of bias), and at review-level (e.g., incomplete retrieval of identified research, reporting bias). | #6 |
| Conclusions | 26 | Provide a general interpretation of the results in the context of other evidence, and implications for future research. | #6 |
| **FUNDING** | | |  |
| Funding | 27 | Describe sources of funding for the systematic review and other support (e.g., supply of data); role of funders for the systematic review. | #6 |

*From:*  Moher D, Liberati A, Tetzlaff J, Altman DG, The PRISMA Group (2009). Preferred Reporting Items for Systematic Reviews and Meta-Analyses: The PRISMA Statement. PLoS Med 6(6): e1000097. doi:10.1371/journal.pmed1000097

For more information, visit: **www.prisma-statement.org**. Page 2 of 2

**Supplementary Table 2.** Quality analyses of the trials included in the systematic review and meta-analysis.

|  | Selection | | | | Comparability | | Outcome | | |  |
| --- | --- | --- | --- | --- | --- | --- | --- | --- | --- | --- |
| Cohort Study | High risk cohort | Low risk cohort | Driven gene tested | TE not present at baseline | Controls for stage | Controls for other factors | TE ascertainment | Duration follow-up | Loss to follow-up | Score out of 9 |
| Chiari R 2020 | 1 | 1 | 1 | 0 | 1 | 0 | 1 | 1 | 1 | 7 |
| Dou F 2018 | 1 | 1 | 1 | 1 | 0 | 0 | 1 | 1 | 1 | 7 |
| Dou F 2020 | 1 | 1 | 1 | 1 | 0 | 0 | 1 | 1 | 1 | 7 |
| Verso M 2015 | 1 | 1 | 1 | 1 | 1 | 0 | 1 | 1 | 0 | 7 |
| Yang S 2020 | 1 | 1 | 1 | 0 | 1 | 0 | 1 | 1 | 1 | 7 |
| Berger N 2014 | 1 | 1 | 1 | 0 | 1 | 0 | 1 | 0 | 1 | 6 |
| Davidsson E 2017 | 1 | 1 | 1 | 0 | 0 | 0 | 1 | 1 | 1 | 6 |
| Delmonte A 2015 | 1 | 1 | 1 | 0 | 1 | 0 | 1 | 1 | 0 | 6 |
| Lee Y G 2014 | 1 | 1 | 1 | 0 | 0 | 0 | 1 | 1 | 1 | 6 |
| Roopkumar J 2020 | 1 | 1 | 1 | 0 | 0 | 0 | 1 | 1 | 1 | 6 |
| Wang J 2019 | 1 | 1 | 1 | 1 | 0 | 1 | 1 | 0 | 0 | 6 |
| Al-Samkari H 2020 | 1 | 1 | 1 | 0 | 0 | 0 | 1 | 1 | 0 | 5 |
| Itchins M 2018 | 1 | 0 | 1 | 0 | 0 | 0 | 1 | 1 | 1 | 5 |
| Ng T L 2019 | 1 | 0 | 1 | 0 | 0 | 0 | 1 | 1 | 1 | 5 |
| Zer A 2017 | 1 | 0 | 1 | 0 | 0 | 0 | 1 | 1 | 1 | 5 |
| Azevedo S 2017 | 1 | 0 | 1 | 0 | 0 | 0 | 1 | 1 | 0 | 4 |
| Leader A 2019 | 1 | 1 | 0 | 0 | 0 | 0 | 1 | 1 | 0 | 4 |
| Muñoz-Unceta N 2020 | 1 | 0 | 1 | 0 | 0 | 0 | 1 | 1 | 0 | 4 |
| Shahzad H 2017 | 1 | 1 | 1 | 0 | 0 | 0 | 1 | 0 | 0 | 4 |
| Xiong W 2020 | 1 | 1 | 1 | 0 | 0 | 0 | 1 | 0 | 0 | 4 |
| Yamazaki S 2013 | 1 | 1 | 1 | 0 | 0 | 0 | 1 | 0 | 0 | 4 |
| Zugazagoitia J 2013 | 1 | 0 | 1 | 0 | 0 | 0 | 1 | 1 | 0 | 4 |
| Zer A 2019 | 1 | 1 | 0 | 0 | 0 | 0 | 1 | 0 | 0 | 3 |
|  | Selection | | | | Comparability | | Exposure | | |  |
| Case-control Study | Case definition | Case selection | Control definition | Control selection | Matched for stage | Matched for other factors | Gene tested | Same method | Non-response rate | Score out of 9 |
| Corrales-Rodriguez L 2014 | 1 | 1 | 1 | 1 | 1 | 1 | 1 | 1 | 1 | 9 |
| Shen Q 2017 | 1 | 1 | 1 | 1 | 1 | 1 | 0 | 0 | 0 | 6 |

**Supplementary Table 3.** Clinical characteristics of patients with driver genes included in the meta-analysis.

|  | *EGFR* (n=2080) | *ALK* (n=1575) | *ROS1* (n=290) | *KRAS* (n=340) | *BRAF* (n=31) | *MET* (n=26) |
| --- | --- | --- | --- | --- | --- | --- |
| Sex (%) |  |  |  |  |  |  |
| Male | 206 (9.9) | 349 (22.2) | 72 (24.8) | 73 (21.5) | 0 (0.0) | 17 (65.4) |
| Female | 363 (17.5) | 438 (27.8) | 113 (39.0) | 79 (23.2) | 0 (0.0) | 9 (34.6) |
| NR | 1511 (72.6) | 738 (46.9) | 105 (36.2) | 188 (55.3) | 31 (100.0) | 0 (0.0) |
| Race (%) |  |  |  |  |  |  |
| White | 175 (8.4) | 202 (12.8) | 31 (10.7) | 85 (25.0) | 0 (0.0) | 0 (0.0) |
| Black | 0 (0.0) | 4 (0.3) | 0 (0.0) | 0 (0.0) | 0 (0.0) | 0 (0.0) |
| Asian | 1473 (70.8) | 261 (16.6) | 120 (41.4) | 127 (37.4) | 26 (83.9) | 0 (0.0) |
| Jewish | 0 (0.0) | 33 (2.1) | 0 (0.0) | 0 (0.0) | 0 (0.0) | 0 (0.0) |
| Arab | 0 (0.0) | 10 (0.6) | 0 (0.0) | 0 (0.0) | 0 (0.0) | 0 (0.0) |
| Other/unknown | 50 (2.4) | 34 (2.2) | 33 (11.4) | 20 (5.9) | 0 (0.0) | 0 (0.0) |
| NR | 382 (18.4) | 1031 (65.5) | 106 (36.6) | 108 (31.8) | 5 (16.1) | 26 (100.0) |
| Smoking history (%) |  |  |  |  |  |  |
| Past/current | 242 (11.6) | 360 (22.9) | 77 (26.6) | 127 (37.4) | 0 (0.0) | 20 (76.9) |
| Never | 325 (15.6) | 715 (45.4) | 165 (56.9) | 24 (7.1) | 0 (0.0) | 6 (23.1) |
| Unknown | 0 (0.0) | 3 (0.2) | 0 (0.0) | 0 (0.0) | 0 (0.0) | 0 (0.0) |
| NR | 1513 (72.7) | 497 (31.6) | 47 (16.2) | 189 (55.6) | 31 (100.0) | 0 (0.0) |
| ECOG (%) |  |  |  |  |  |  |
| 0-1 | 180 (8.7) | 547 (34.7) | 152 (52.4) | 25 (7.4) | 0 (0.0) | 24 (92.3) |
| 2-3 | 182 (8.8) | 183 (11.6) | 86 (29.7) | 107 (31.5) | 0 (0.0) | 2 (7.7) |
| NR | 1718 (82.6) | 821 (52.1) | 52 (17.9) | 208 (61.2) | 31 (100.0) | 0 (0.0) |
| Histopathology (%) |  |  |  |  |  |  |
| Adenocarcinoma | 1009 (48.5) | 1157 (73.5) | 222 (76.6) | 271 (79.7) | 5 (16.1) | 23 (88.5) |
| Other/unknown | 7 (0.3) | 64 (4.1) | 21 (7.2) | 7 (2.1) | 0 (0.0) | 3 (11.5) |
| NR | 1064 (51.2) | 354 (22.5) | 47 (16.2) | 62 (18.2) | 26 (83.9) | 0 (0.0) |
| Stage (%) |  |  |  |  |  |  |
| I | 38 (1.8) | 19 (1.2) | 2 (0.7) | 11 (3.2) | 0 (0.0) | 0 (0.0) |
| II | 18 (0.9) | 8 (0.5) | 1 (0.3) | 9 (2.6) | 0 (0.0) | 0 (0.0) |
| III | 25 (1.2) | 111 (7.0) | 23 (7.9) | 23 (6.8) | 0 (0.0) | 0 (0.0) |
| IV | 416 (20.0) | 920 (58.4) | 161 (55.5) | 108 (31.8) | 0 (0.0) | 26 (100.0) |
| III-IV | 356 (17.1) | 58 (3.7) | 5 (1.7) | 77 (22.6) | 5 (16.1) | 0 (0.0) |
| Unknown | 0 (0.0) | 6 (0.4) | 13 (4.5) | 0 (0.0) | 0 (0.0) | 0 (0.0) |
| NR | 1227 (59.0) | 453 (28.8) | 85 (29.3) | 112 (32.9) | 26 (83.9) | 0 (0.0) |
| Treatment (%) |  |  |  |  |  |  |
| TKI | 379 (18.2) | 1038 (65.9) | 64 (22.1) | 3 (0.9) | 0 (0.0) | 0 (0.0) |
| Chemotherapy | 127 (6.1) | 349 (22.2) | 50 (17.2) | 125 (36.8) | 0 (0.0) | 0 (0.0) |
| Surgery | 138 (6.6) | 19 (1.2) | 0 (0.0) | 18 (5.3) | 0 (0.0) | 0 (0.0) |
| Radiotherapy | 9 (0.4) | 4 (0.3) | 0 (0.0) | 0 (0.0) | 0 (0.0) | 0 (0.0) |
| No treatment | 5 (0.2) | 2 (0.1) | 0 (0.0) | 0 (0.0) | 0 (0.0) | 0 (0.0) |
| Other/unknown | 14 (0.7) | 7 (0.4) | 15 (5.2) | 4 (1.2) | 0 (0.0) | 0 (0.0) |
| NR | 1394 (67.0) | 298 (18.9) | 153 (52.8) | 170 (50.0) | 31 (100.0) | 26 (100.0) |
| Oncogene identification (%) | | | | | | |
| RT-PCR or NGS | 1608 (77.3) | 261 (16.6) | 187 (64.5) | 313 (92.1) | 31 (100.0) | 10 (38.5) |
| Fluorescence in situ | 0 (0.0) | 208 (13.2) | 37 (12.8) | 0 (0.0) | 0 (0.0) | 16 (61.5) |
| Medical record | 421 (20.2) | 116 (7.4) | 0 (0.0) | 27 (7.9) | 0 (0.0) | 0 (0.0) |
| Crizotinib prescriptions | 0 (0.0) | 321 (20.4) | 0 (0.0) | 0 (0.0) | 0 (0.0) | 0 (0.0) |
| Unknown | 0 (0.0) | 0 (0.0) | 2 (0.7) | 0 (0.0) | 0 (0.0) | 0 (0.0) |
| NR | 51 (2.5) | 669 (42.5) | 82 (28.3) | 0 (0.0) | 0 (0.0) | 0 (0.0) |

Abbreviation: NR, not reported; ECOG, Eastern Cooperative Oncology Group; TKI, tyrosine kinase inhibitors; RT-PCR, reverse transcriptase polymerase chain reaction; NGS, next-generation sequencing.

# Supplementary Figures

#
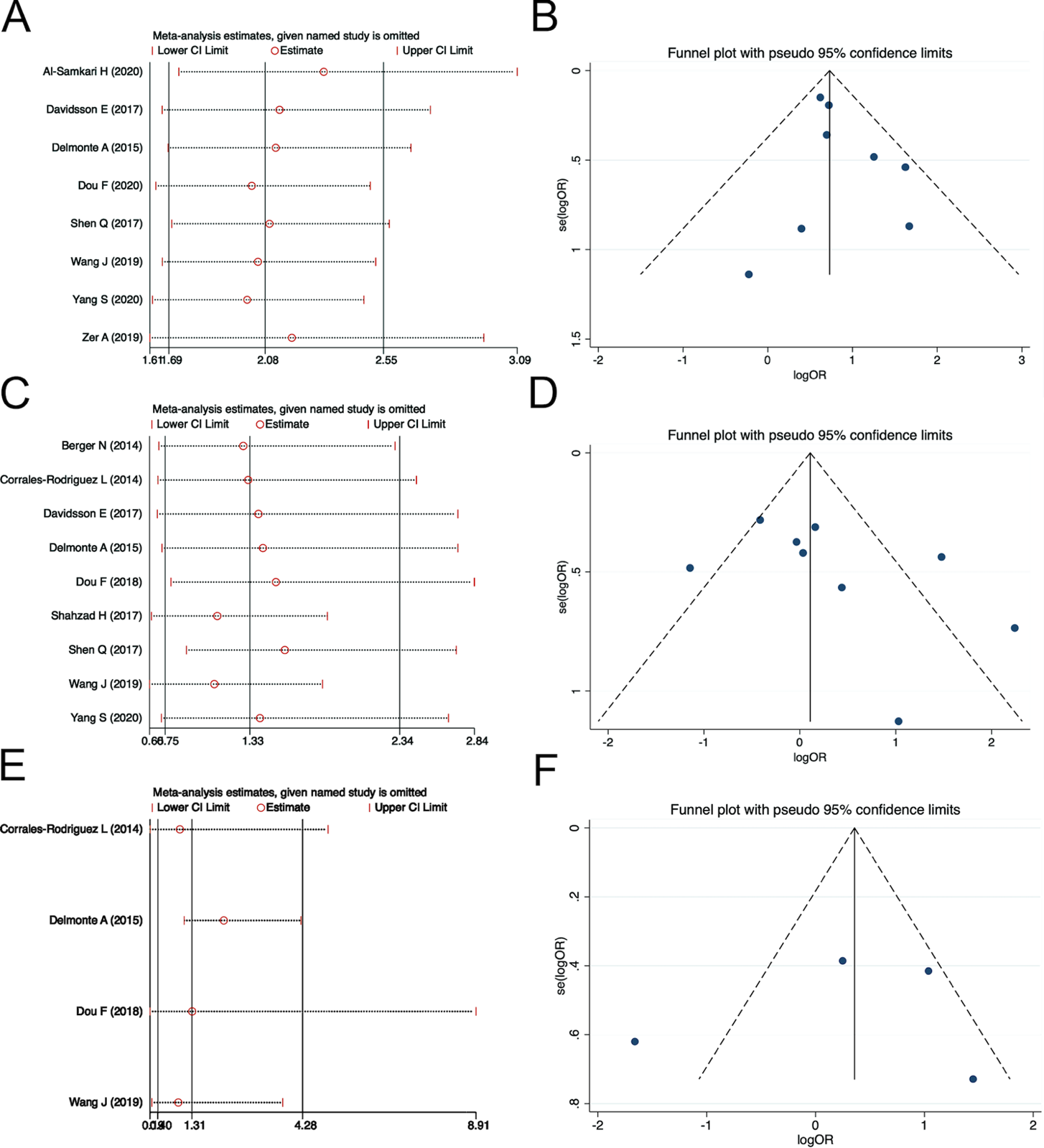


**Supplementary Figure 1.** Sensitivity analyses and funnel plots according to driver genes on studies included for risk estimates of VTE. (A) Sensitivity analysis of studies on *ALK* rearrangements; (B) The funnel plot of studies on *ALK* rearrangements; (C) Sensitivity analysis of studies on *EGFR* mutations; (D) The funnel plot of studies on *EGFR* mutations; (E) Sensitivity analysis of studies on *KRAS* mutations. (F) The funnel plot of studies on *KRAS* mutations.

**
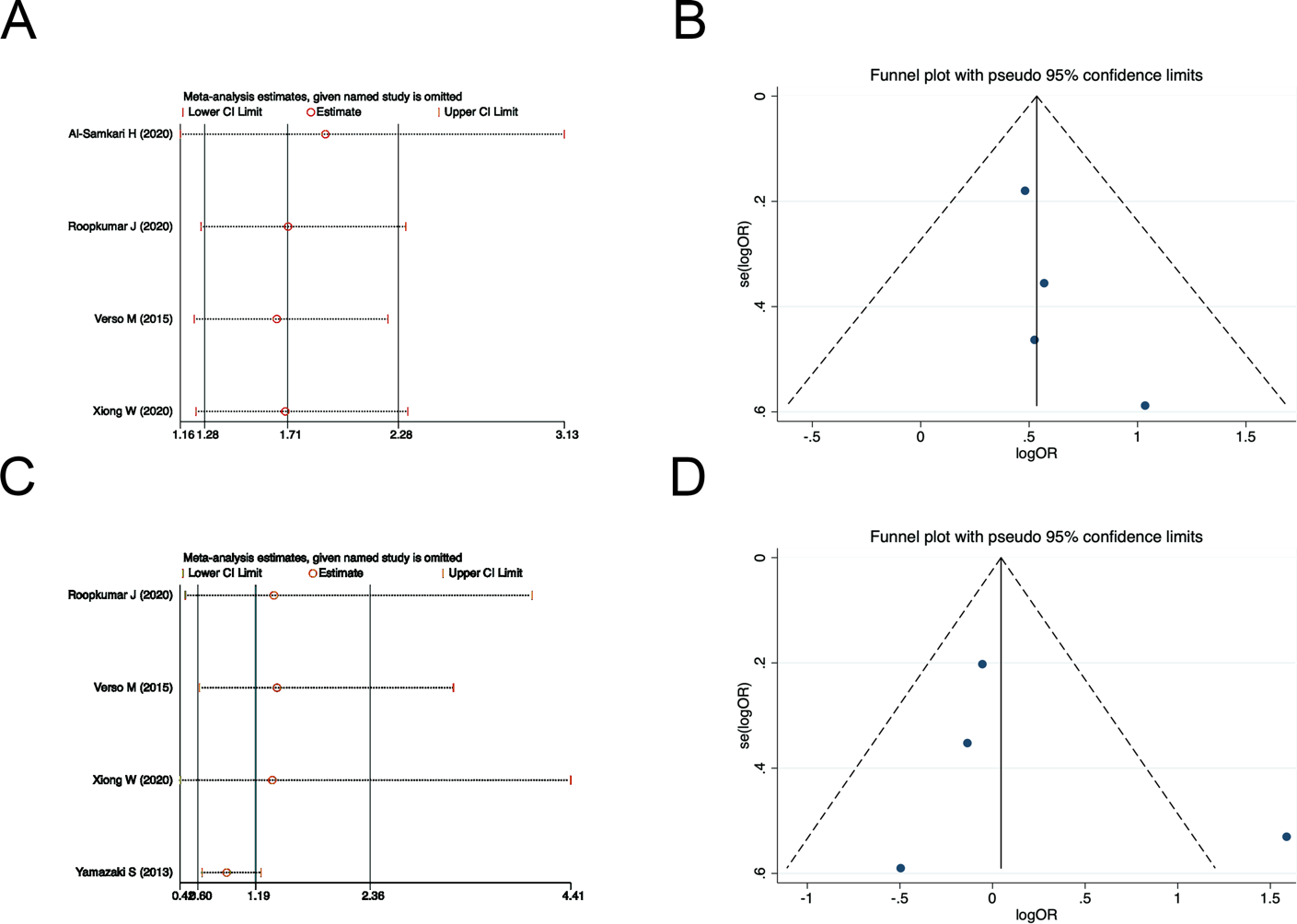
**

**Supplementary Figure 2.** Sensitivity analyses and funnel plots according to driver genes on studies included for risk estimates of PE. (A) Sensitivity analysis of studies on *ALK* rearrangements; (B) The funnel plot of studies on *ALK* rearrangements; (C) Sensitivity analysis of studies on *EGFR* mutations; (D) The funnel plot of studies on *EGFR* mutations.
